# Ethical Governance Strategies for the Responsible Innovation of Neurotechnologies: A Scoping Review

**DOI:** 10.1007/s11673-025-10440-9

**Published:** 2025-11-11

**Authors:** Liam J. Robertson, Nathan L. Higgins, Moritz J. Maier, Adrian Carter, John G. Gardner

**Affiliations:** 1 School of Psychological Sciences, Monash University, Melbourne, Australia; 2 Monash Bioethics Centre, School of Philosophical, Historical and Indigenous Studies, Monash University, Melbourne, Australia; 3 Centre for Responsible Research and Innovation at the Fraunhofer IAO, Berlin, Germany; 4 School of Social Sciences, Monash University, Melbourne, Australia

**Keywords:** Neurotechnology, Ethics, Scoping review, Responsible innovation, Guidelines, Framework, Governance

## Abstract

Following the recent surge in neurotechnology innovation and commercial investment, numerous academic bodies, government bodies, multilateral organizations, and industry leaders have produced ethical guidelines to govern neurotechnology innovation. Many highlight the need for new regulations to protect the rights and welfare of vulnerable individuals, while others warn about unnecessarily impeding innovation that provides urgent treatments to intractable conditions. Consensus on appropriate governance of neurotechnological innovation remains elusive. To fill this gap, we conducted a scoping review of the academic literature concerning the governance of neurotechnology development, identifying the ethical issues addressed, highlighting gaps or underdeveloped areas of neurotechnology governance, and the country in which they were developed. Fifty-one academic articles from the peer-reviewed literature were extracted, selecting those that referred to neurotechnologies and presented normative ethical guidelines or frameworks for their governance. We identified six common ethical themes (justice, beneficence and nonmaleficence, privacy and brain data, autonomy and informed consent, and, identity, dignity, and moral status) and six governance strategies proposed to address these themes (social responsibility and accountability, interdisciplinary collaboration, public engagement, scientific integrity, epistemic humility, legislation and neurorights). Discussions surrounding these themes lacked adequate consideration of diverse viewpoints, such as from the Global South, and were often underdeveloped, lacking both practical specificity and guidance to help developers balance competing priorities. Specifically, animal ethics and binding governance approaches were not adequately addressed, while neurorights were given undue consideration. Future guidelines should engage with these areas to aid in the development of more comprehensive and specific governance documents.

**Supplementary Information:**

The online version contains supplementary material available at 10.1007/s11673-025-10440-9.

## Introduction

Neurotechnologies are a class of technologies that directly manipulate, monitor, or interact with the brain and central nervous system (Müller and Rotter 2017). They include recording or sensing technologies, such as electroencephalography (EEG) and functional magnetic resonance imaging (fMRI), modulating/stimulating devices, such as deep brain stimulation (DBS), and technologies that combine both sensing and stimulation to control the nervous system, such as brain-computer interfaces (BCI) and closed-loop DBS.

Neurotechnology has a critical role in neuroscience research and is a major enabler of translational neuroscience; the utilizing of neuroscience research for the diagnosis and treatment of neurological and psychological disorders (Garden et al. 2019). Advances in neurotechnology are essential to overcome key challenges in neuroscience research, namely: (a) reliably measuring neuronal activity; (b) mapping neuronal activity onto a comprehensive functional index of the brain; and (c) understanding or decoding the brain by deciphering large amounts of brain data (Abbott 2013). The hope is that overcoming such challenges will enable the development of much needed preventative, diagnostic, and therapeutic solutions that will address the burden of neurological and psychological disorders and improve the lives of millions of people worldwide (Feigin et al. 2017).

There has been a rapid growth in neurotechnology development, supported by substantial investments from public initiatives, notably the U.S. BRAIN initiative and European Human Brain Project, and private companies, including Google and Neuralink (U.S. National Institutes of Health 2023; Human Brain Project 2023; Neurotech Reports 2018). These advancements, however, raise significant ethical concerns, particularly around privacy of personal information, discrimination when used by third parties, autonomy and responsibility for behaviour resulting from neurotechnological intervention, and biases in how the risks and benefits are distributed throughout society (Yuste et al. 2017). Fears include the potential for third party access to personal “brain data,” and the impact of neuromodulation on personality, decision-making, and responsibility for these choices (Ienca et al. 2022; Goering et al. 2021). These concerns are magnified by the currently sparse legal safeguards protecting the expansion of neurotechnologies into non-therapeutic commercial applications marketed directly to consumers (Garden et al. 2019).

These ethical concerns have prompted various regulatory and government organizations (Garden et al. 2019; Greely et al. 2018) and researchers (Yuste et al. 2017; Amadio et al. 2018) to develop ethical frameworks that govern the development and use of neurotechnology. Despite the emergence of these frameworks, there remains no explicit consensus among these various ethical governance strategies. These frameworks vary in their scope and recommendations, often resulting in confusion and inconsistent adherence among researchers and developers (Pham et al. 2023). They have also been criticized as lacking specificity, making practical implementation difficult (O’Shaughnessy et al. 2023). Ethical guidelines that are unclear, impractical, or misaligned with the realities of research and clinical settings can hinder the ability and willingness of research groups and stakeholders to follow them effectively. There is also a need for these frameworks to reflect a broader range of perspectives, particularly from underrepresented groups (Amadio et al. 2018; Matshabane et al. 2022). International bodies have begun to develop new recommendations and guidelines, most notably UNESCO, which released a draft report for the governance and innovation of neurotechnology (UNESCO 2024). It is important that these reports recognize the guidance of existing guidance documents and consider diverse academic viewpoints that have considered these issues over many years and in different contexts (Bublitz 2023). Whilst policy documents are important, researchers, developers, and clinicians might have difficulty implementing the current corpus of ethical guidelines and frameworks in their current state.

We conducted a scoping review to synthesize current ethical frameworks and identify current gaps that may direct future tools governing neurotechnology innovation. We also identified stakeholders that were involved in their development and the degree of diversity and convergence regarding the types of ethical issues identified and the strategies that are proposed to manage them. In doing so, we addressed the following research questions: (1) What ethical issues and normative strategies are addressed in current guidelines governing neurotechnologies? (2) What are the ethical or normative gaps in the current guidelines? and (3) Where and when were the guidelines published? These findings can inform the development of more appropriate, participatory, and practical guidelines to ensure the ethical development and application of neurotechnologies.

## Methods

A scoping review was conducted using Levac et al. (2010) revised version of the approach originally introduced by Arksey and O’Malley (2005). This method allows for research findings to be identified and summarized and for key themes and gaps in the literature to be examined. This method entailed the following steps:

### Identifying the Research Questions

The research questions listed above were developed by the team to address the key aim of the study, namely to outline and critically analyse the current ethical guidelines governing neurotechnology development, identifying key stakeholders, and proposing recommendations for creating more effective, participatory, and practical frameworks.

### Identifying Relevant Studies

A systematic literature search was conducted to identify journal articles providing ethical guidelines or frameworks for neurotechnology development. The databases PsycINFO, Ovid MEDLINE, Scopus, ISI Web of Science, and ProQuest were chosen for their broad and comprehensive coverage. Findings from the study were reported according to the PRISMA Extension for Scoping Reviews (PRISMA-ScR) guidelines, including inclusion/exclusion, data extraction, and analysis and reporting (Tricco et al. 2018).

**Fig. 1:**
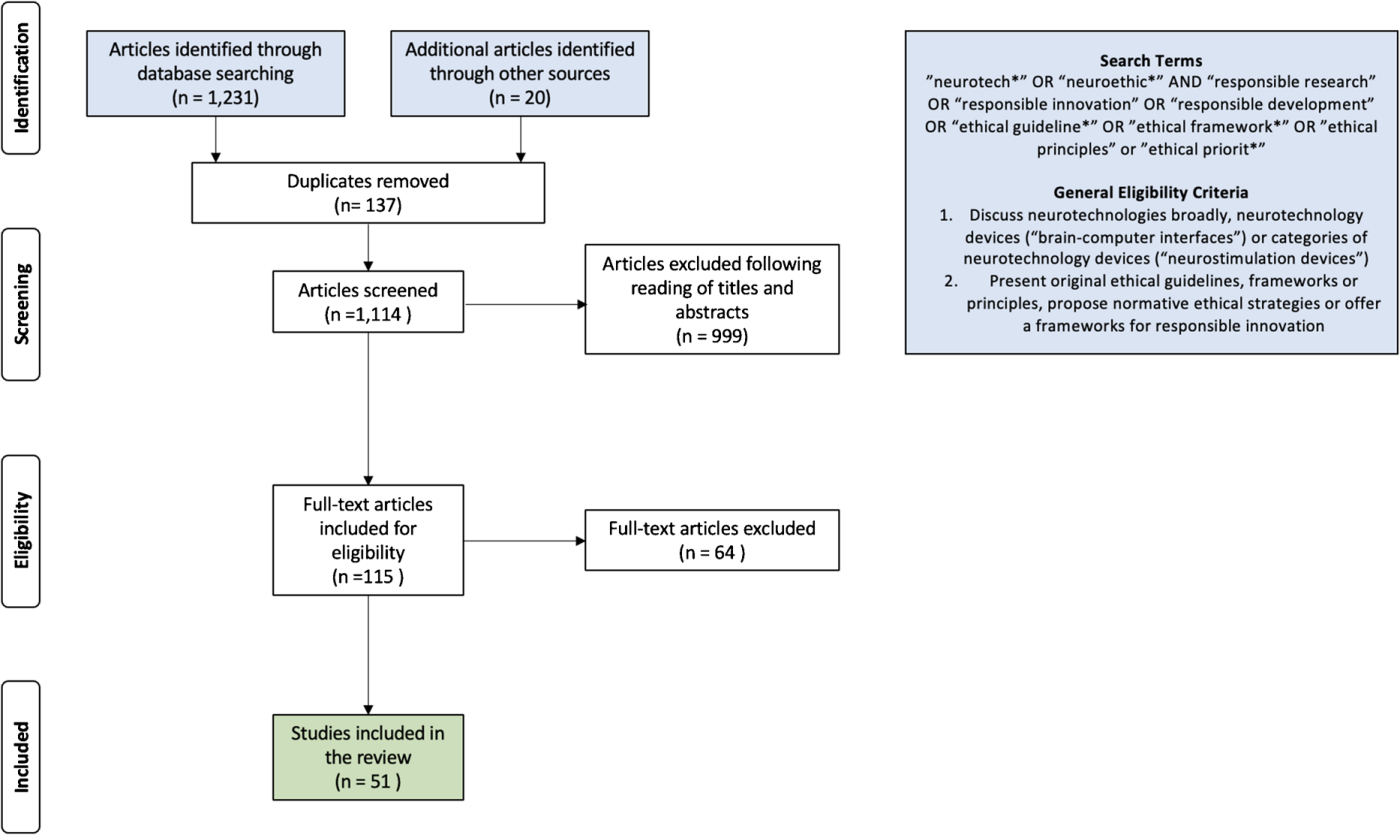
PRISMA-based flowchart of retrieval process, search terms and general eligibility criteria

A search was conducted on June 14, 2023 using keywords related to neurotechnology, neuroethics, and associated ethical frameworks and guidelines (see, Fig. 1). The strategy was tested and iterated before data collection to maximize the inclusion of relevant papers. Keywords were developed by scanning the literature for terms describing neurotechnology governance. These terms were sequentially included to ensure each new keyword added relevant articles. The search results were cross-checked against known articles to ensure the search accuracy. Reference chaining was used to identify additional articles not retrieved in the final search strategy. The search terms and a flow chart of this retrieval process can be seen in Fig. 1.

### Study Selection

Records underwent three stages of screening: (i) title, (ii) abstract, and (iii) full text, to ensure they met the inclusion criteria. Quality assurance was maintained at each stage with a random selection of 10 per cent of the articles dual screened by co-authors achieving 80 per cent consensus (Barnett et al. 2018). Any disagreements at the final screening stage were resolved through discussions with all co-authors until consensus was reached.

Articles were included from any year if they met the following criteria: (i) written in English; (ii) mentioned neurotechnologies in general, specific neurotechnologies (e.g., DBS), categories of neurotechnologies (e.g., stimulation devices), or research into neurological disorders treatable with neurotechnologies; (iii) presented original ethical guidelines or frameworks for responsible neurotechnology innovation. Articles were excluded if they only provided commentaries of existing guidelines or did not provide normative guidance for the ethical governance of neurotechnology.

### Charting the Data

Data was extracted on ethical issues and proposed strategies to address them. Bibliographic data relevant to the study aims and research questions were also collected, including year of publication, country of origin, and stakeholders consulted (e.g., patients, clinicians, end-users, people with lived experience, marginalized communities). Ethical issues and governance strategies were coded inductively and iteratively, allowing themes to emerge from the data (Creswell et al. 2007). Data were categorized into two major areas: ethical themes (e.g., justice) and governance strategies (e.g., public engagement), and content analysis was conducted using NVivo12 software, while demographic information and publication year were organized in an Excel table. These codes were reviewed by the research team and 10 per cent of the articles were double coded.

### Collating, Summarizing, and Reporting the Results

A content analysis was conducted to identify broad and specific categories from the data (Hsieh and Shannon 2005). The process included: (i) reading the data as a whole, (ii) reading data word for word to code language describing ethical issues or governance strategies, (iii) identifying categories from the codes, and (iv) generalizing categories across all extracted codes. Articles are presented narratively, supported by descriptive summaries of key themes from each article (Arksey and O’Malley 2005). This analysis is supplemented with diagrammatic and tabular presentations of the bibliographic contents, addressing who was involved in the development of these documents (e.g., country of origin and stakeholders consulted in guideline development).

### Consultation

Our findings were externally reviewed through consultations with six experts in the field, including a clinical psychologist, TMS scientist, neuroimaging scientist, ethicist, legal expert, and a person with lived experience. Consultants were asked to read a late-stage draft of the study results and comment on the accuracy, clarity, and comprehensiveness of the findings. Based on their feedback, we revised the manuscript to refine our discussion and explanation of the ethical issues and suggestions for addressing them. Their expertise also identified ethical issues not adequately considered in the existing literature**.**

## Results

**Fig. 2:**
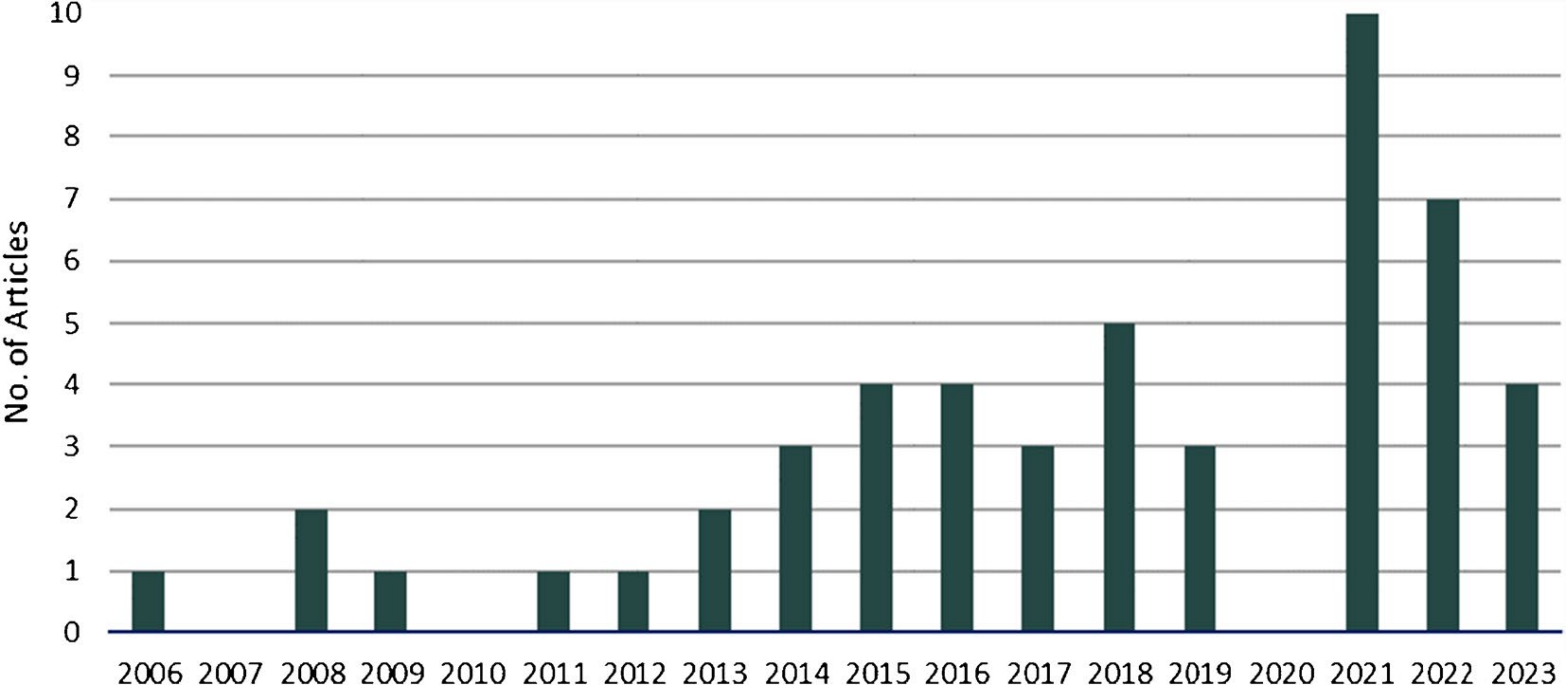
Articles published by year

The screening process identified fifty-one articles containing ethical frameworks and guidelines regarding the research, innovation, and use of neurotechnologies. Data revealed a substantial increase over time in the number of publications, with 63 per cent being published after 2018, as shown in Fig. 2.

**Fig. 3:**
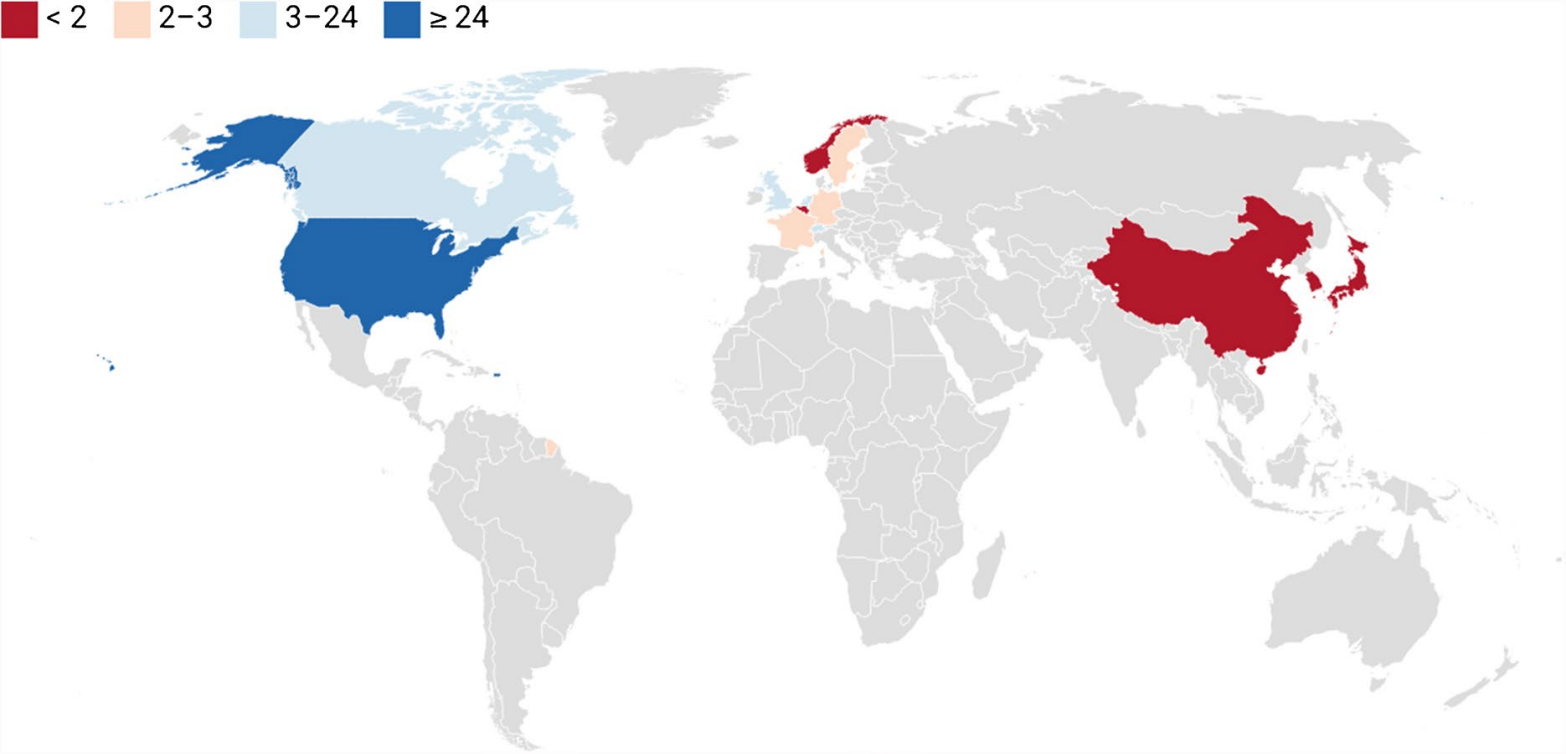
Geographic distribution of first authors by number of articles published

The geographic distribution (see, Fig. 3) showed greater representation in economically developed countries, with the United States (n = 24, 47 per cent) accounting for nearly half of all sources, followed by Canada (n = 5), the United Kingdom (n = 4), Switzerland, and the Netherlands (each n = 3). China was the only country represented from the Global South. Significantly, no articles reported the involvement of diverse stakeholders, such as people with lived experience or those from marginalized communities.

Six overarching ethical themes were identified, encapsulating the ethical issues that emerged from the dataset. In order of frequency, they were: Justice, Beneficence and Non-Maleficence, Privacy and Brain Data, Agency and Autonomy, Informed Consent, Identity, Dignity and Moral Status (Table 1). Seven governance strategies or approaches to address these ethical themes emerged: Social Responsibility and Accountability, Interdisciplinary Collaboration, Public Engagement, Scientific Integrity, Epistemic Humility, Legislation and Neurorights (Table 2). A detailed thematic interpretation of the documents is provided in the following. For ease of reading, references are assigned a number corresponding to an index (Supplementary Material 1). For a summary of these results, see (Table 3).

**Table 1:** Frequency of ethical themes mentioned in the articles

Ethical Themes	No. of Articles
Justice	25
Beneficence and Non-Maleficence	22
Privacy and Brain Data	20
Agency and Autonomy	17
Informed Consent	17
Identity, dignity, and moral status	12

**Table 2:** Frequency of governance strategies mentioned in the articles

Governance strategies	No. of Articles
Social Responsibility and Accountability	25
Interdisciplinary Collaboration	25
Public Engagement	19
Scientific Integrity	15
Epistemic Humility	15
Neurorights	7
Legislation	6

**Table 3:** Summary of the coded themes and their corresponding supporting articles

**Ethical themes**	**Ethical issues**	**Supporting articles**
Justice	Affordability and accessibility concerns	8, 12, 18, 20, 23, 34, 40, 48,
	Concerns about enhancement tech	9, 11, 16, 24, 26, 36, 37
	Health inequalities and vulnerable populations	6, 11, 18, 23
	Fairness in academic/work environments	24, 36
	Unjust distribution of research benefits and risks	7, 9, 15, 16, 22, 23, 36
	Stigmatization and discrimination (neural data)	9, 34, 44, 48,
	Prejudicial impacts on employment and insurance	9, 34, 48
	Avoid replicating cultural/systemic bias	9, 15, 24, 35
	Ensuring representative datasets	6, 7, 17, 51
	Approaches to combat bias in research	2, 6, 16, 35, 40
Beneficence and Non-Maleficence	Balancing risks and benefits	13, 15, 18, 28
	Ongoing costs of care	9, 12, 15, 18, 25, 40
	Prioritizing further research to evaluate harms/benefits	10, 42
	Device safety concerns	22, 23, 33, 36, 47, 51
	Risks of invasive tech (DBS, BCIs)	8, 33, 40, 41, 49
	Biocompatibility concerns	40
	Risks of relying on obsolete technologies	9, 40
	Off-label neuroenhancement risks	37
	Reliance on neuroenhancement modifications	4
Privacy and Brain data	Protecting privacy of brain data	2, 17, 20, 22, 23, 24, 35, 40, 44, 45, 46, 47
	Consent issues with brain data	16, 35, 47
	Brain profiles predicting behaviour	2, 16, 20, 23, 24, 34, 35, 46, 48
	Coerced collection of brain data	35, 47
	Security lapses and hacking	23, 35, 40, 41, 46, 47
	Safeguarding participants’ data	23, 46, 47
	Recommendations for encryption and anonymization	2, 17, 20, 23, 35, 46
	Proposals for legislative updates	2, 47
	Mandating user agreements for consent	16, 35, 47
Agency	Modulation of neural activity undermining agency	1, 4, 6, 7, 8, 16, 20, 22, 23, 24, 35, 37, 40, 41, 45, 47, 49
	AI algorithms influencing decisions	45
	Blurring human/computer-driven actions	23, 35
Informed consent	Informed consent as ongoing process	7, 10, 13, 15, 16, 18, 23
	Accurate information for patients/participants	10, 23, 35
	Assessing capacity for consent	10, 23
	Realistic expectations of research	15, 18, 23
	Impaired decision-making capacity	10, 13, 15, 23
	Consent issues with brain data sharing	6, 20, 24, 35, 47
	Commercial technology consent concerns	16, 18, 35, 47
	Incidental findings disclosure	1, 7, 21, 24, 25, 34, 48
	Clear information with visual aids	23, 35, 47
Identity, dignity and moral status	Impact of neurotech on identity	15, 16, 23, 47, 49
	Concerns over agency and psychological continuity	16, 24, 41, 43
	Blurring line between human/machine cognition	24, 41, 45
	Threats to human dignity and capacities	4, 24, 36, 45, 47
**Governance strategies**	**Normative approaches**	**Supporting Articles**
Social responsibility and accountability	Promote social good	6, 8, 9, 10, 15, 16, 17, 21, 24, 29, 35, 38, 39, 41, 47, 48, 50, 51
	Proactive responsibility	6, 9, 14, 21, 24, 29, 38, 45, 47, 48, 51
	Discourage enhancement research	10, 11, 15, 21, 23
	Regulate enhancement research	24
	Cultural and social shifts	21, 27, 45
	Promote stewardship	51
	Accountability across hierarchy	13, 43
	Develop professional ethical codes	16, 17, 50
	Foster responsible culture	6, 9, 38
	Support and incentives	6, 24, 35
Interdisciplinary collaboration	Interdisciplinary researcher collaboration	5, 13, 15, 17, 18, 24, 29, 41, 45
	Stakeholder engagement	6, 24, 27, 38, 39, 40, 42, 45, 47, 51
	Culturally inclusive dialogues	24, 27, 39, 47, 51
	Diverse perspectives on equality and justice	24, 27, 39, 47, 51
	Leverage external ideas	6, 29, 45
	Local to international collaborations	19, 25, 31, 35, 43, 48, 51
	Internationally accepted governance	6, 16, 24, 47
Public engagement	Responsibility to communicate findings to the public	15, 21
	Engage public in dialogues on societal impact	5, 6, 16, 17, 21, 22, 23, 40, 41, 51
	Public voice in policy decisions	3, 16, 24, 31, 39
	Empirical methods to assess public views	24
	Building community trust and social licence	6, 13, 23, 51
	Educating public on ethical issues	6, 16, 22, 23, 41, 45, 50
	Training for specialists and health professionals	6, 13, 16, 18, 24
Scientific Integrity	Rigorous scientific practices	13, 15, 23, 47
	Open science and open data sharing	3, 4, 6, 18, 21, 29, 42, 45
	Careful data collection and management	15, 18
	Reproducibility	21, 47
	Balanced reporting of results	5, 42
	Avoidance and disclosure of conflicts of interest	12, 37
Epistemic humility	Damage from unrealistic expectations	2, 15, 40, 43, 48
	Balanced portrayal of technology benefits	8, 11, 17, 21, 23, 29, 47, 49
	Clarifying limits of scientific knowledge	21, 49
	Anticipating and dispelling false narratives	5, 34
	Identifying sensitive public issues	23
	Public disillusionment from misconceptions	34, 40
	Communicating alternative, non-technological solutions	8, 11
Legislation	Necessity of legislation for safe and responsible use	16, 40
	Legislation potentially hindering innovation	34, 51
	Standardization	5, 42
	Broader legislative proposals (e.g., international conventions)	16
Neurorights	Neurorights to protect brain data and neurological manipulation	16, 20, 30, 35
	Neurorights addressing brain data privacy	20, 30, 35, 44, 47
	Concerns about agency and identity	16, 20, 30
	Philosophical concerns: free will and freedom of thought	20, 30, 47
	Right to be shielded from harmful mental manipulations	30, 35, 47
	Arguments against narrow focus on neurorights	40
	Neurorights enhancing data protection	16, 30, 35, 47

### Ethical Themes

#### Justice

Justice is largely expressed as fairness; the notion of giving to each what is due (Rawls 1971). Many articles expressed concerns about the affordability and accessibility of neurotechnologies (8, 12, 18, 20, 34, 40, 48, 23), and specifically enhancement neurotechnologies (9, 11, 16, 24, 26, 36, 37), including for vulnerable populations and marginalized groups (6, 18), with calls to avoid exacerbating existing health inequalities (11, 23). Enhancement technologies, like transcranial direct current stimulation (tDCS) for improving memory, also raised concerns about fairness and potential cheating in academic or work environments (24, 36). In a research context, there was discussion of historically unjust distribution of research benefits (7, 9, 15, 16, 22, 23, 36) and risks (7, 9, 15, 16, 22, 23, 36), particularly amongst historically marginalized groups.

Stigmatization and discrimination related to neural data and brain profiles were noted (9, 34, 44, 48), raising fears about prejudicial impacts on employment (48) and insurance (9, 34, 48). Concerns included constructing social categories and stereotypes from neural data (2, 24). Sources also emphasized that current cultural and systemic bias (gender, race, ability etc.) should not be replicated in neurotechnology research design and analysis (9, 15, 24, 35). Ensuring representative neural datasets and inclusivity of historically excluded groups (6, 7, 17, 51) was viewed as essential to eliminate bias and enhance functionality for diverse populations (35, 40, 47). Approaches to combat bias in research included incorporating diverse perspectives during the early stages of the research process (2, 6, 16, 35, 40), bias checklists (35), unconscious bias training (16, 35), and community engagement through focus groups (16, 35, 40).

#### Beneficence and Non-Maleficence

Most literature discussed beneficence and non-maleficence as critical considerations in the treatment of patients and research participants. These discussions included the need to balance the risk of harms and benefits (13, 15, 18, 28), account for the ongoing costs of care (9, 12, 15, 18, 25, 40), and prioritize further research to evaluate the harms and benefits (10, 42). Concerns about non-maleficence predominantly focused on device safety (22, 23, 33, 36, 47, 51), such as the unknown or unforeseen risks of invasive neurosurgical technologies, such as DBS (33, 49), BCIs (8, 40, 41), and future enhancement technologies (35, 36). The biocompatibility of implanted technologies (40) and the risks of trial participants relying on obsolete and unsupported technologies once a trial has concluded (9, 40) were important considerations. Issues involving enhancement technologies also include the challenges of prescribing neuroenhancement for off-label use without adequate clinical studies on their safety (37) and the risk of individuals becoming excessively reliant on these modifications (4).

#### Privacy and Brain Data

Many articles referred to the importance of protecting privacy (2, 17, 20, 22, 23, 24, 35, 40, 44, 45, 46, 47), a right to privacy (30, 35), and the adherence of neural data to established data confidentiality standards (7, 21, 22, 46). Articles emphasized that brain data extraction bypasses the executive control mechanisms we use to withhold information or consent to its use (30, 47). Others stressed that brain data included unexecuted behaviour, like inner speech (23, 35, 47) for which there is currently little legal protection (47).

Concerns over brain data are categorized into four main areas. First, inadequate informed consent related to the sharing and future use of brain data, paralleling the issues seen with complex digital terms and conditions (16, 35, 47). Second, advances in machine learning raise uncertainties about how much personal information may be derived from brain data in the future, including “brain profiles” that predict future behaviour or the development of mental or neurological disorders (2, 16, 20, 23, 24, 34, 35, 46, 48), with risks of this data being misused or sold to third parties due to regulatory gaps (2, 9, 24, 40, 47). Third, the coerced collection and sharing of brain data was raised, especially in contexts involving significant power disparities, such as in workplaces and education (35, 47). Fourth, privacy breaches through security lapses and hacking were a concern (35,47), particularly for BCIs that decode neural data into thoughts that could be read by malicious parties (23, 35, 40, 41, 46, 47). Attention was given to safeguarding study participants’ data (20, 23), with concern about recent algorithms that can re-identify participants in neuro-imaging datasets that were thought to be de-identified, through a process known as “MRI fingerprinting” (23, 46, 47).

In protecting privacy, articles highlighted some ethical recommendations such as robust encryption (2, 17, 20, 23, 35), anonymizing datasets (2, 20, 46), limiting data collection and access (2, 20, 35, 46), and enhancing security measures (46). Further, suggestions to update consumer device and labour laws aimed to prevent unauthorized corporate access to personal data (47). Additionally, some proposed legislation aimed to ban data re-identification (2), mandate data destruction post-use (20, 44), restrict brain data sales (35), and prevent unauthorized corporate access to personal data (47). Mandating device user agreements so that organizations cannot share personal data was suggested to safeguard consent (16, 35, 47).

#### Agency and Autonomy

A prevalent ethical concern is the potential for neurotechnologies to modulate neural activity in ways that undermine user agency (1, 4, 6, 7, 8, 16, 20, 22, 23, 24, 35, 37, 40, 41, 45, 47, 49). Agency was specifically defined as the freedom to choose one’s actions (23,35). This concern is heightened with technologies like BCIs, where AI algorithms may externally influence decisions, blurring the line between human and computer-driven actions (45). These issues of agency and autonomy are central to broader ethical discussions on informed consent, identity, human dignity, and moral status. We discuss these manifestations next.

#### Informed Consent

Informed consent was highlighted as a key theme in neuroscience and neurotechnology research (7, 10, 13, 15, 16, 18, 23). Consent was emphasized as an ongoing process (10, 13) whereby researchers and clinicians need to provide patients and participants with accurate information (10, 23, 35), continually assess participants' capacity for consent (10, 23), and set realistic expectations about the likely harms and benefits of the research (15, 18, 23). Concern was expressed over neurotechnology research in patients who may have impaired decision-making capacity (10, 13, 15, 23). Treatments may also involve manipulating the neural processes needed for consent (23, 35), such as a person’s identity (13, 16, 35), which may not always be made clear during the consent processes. Concern over the collection and sharing of brain data was also prevalent (6, 20, 24, 35, 47), with particular concern over consent processes in commercial technologies due to asymmetric information between users and commercial owners (16, 18, 35, 47). Informing patients about the potential for incidental findings (1, 7) and considering the risks and benefits to participants of disclosing these findings (1, 7, 21, 24, 25, 34, 48), were also highlighted. Articles stressed the importance of adequately informing the public (35), avoiding exaggeration of benefits (23) or minimization of harms (13), and using clear and complete information (23, 35, 47) with simple wording and visual aids (35).

#### Identity, Dignity, and Moral Status

Many sources discuss the potential impact of neurotechnologies on identity (47), citing examples like DBS (15, 16, 23) and TMS (49), which have been found to alter critical aspects of personality in some users. These technologies, often used for treating psychiatric illnesses such as Parkinson’s disease, OCD, and depression, can unpredictably affect one’s sense of identity (35).

The capacity of these technologies to alter agency and psychological continuity, experiencing oneself as the same person over time (43), raises questions about personal responsibility (16, 24, 41, 43). For instance, BCIs using AI or machine learning might blur the line between human and non-human cognition and behaviour (24, 41, 45). Furthermore, enhancing performance or cognition through neurotechnologies is seen as a fundamental threat to human capacities (24), dignity (36), and the essence of being human (4, 45, 47).

### Governance Strategies

The above section highlighted the ethical themes and related ethical recommendations. However, most articles proposed broader systemic changes in disciplinary, institutional, or public policy. They provided frameworks for responsible innovation, ranging from soft governance, such as professional standards and guidelines, to hard governance, including legislative changes with punitive consequences for non-compliance. These governance strategies are detailed below.

#### Social Responsibility and Accountability

Most articles emphasized the social responsibilities of neurotechnology developers to promote social good (6, 8–10, 15, 16, 17, 21, 24, 29, 35, 38, 39, 41, 47, 48, 50, 51). Articles highlighted that social responsibility should be proactive, anticipating the direct and indirect outcomes of research (6, 21, 24, 29, 38, 45, 47, 48, 51), including potential dual uses in military settings (9, 14). Some suggested that research into non-healthcare related uses, such as enhancement, should be discouraged (10, 11, 15, 21, 23) or carefully regulated (24).

Concerns were raised about the lack of reflection on the social and cultural shifts that could occur if neurotechnologies become widely available (21, 27, 45). Several articles called on companies and developers to promote stewardship (51), stating they need to be held accountable for the indirect implications of their products (16, 48), and this accountability should extend to all levels in an organizational hierarchy (13, 43). To ensure social responsibility, articles recommended developing professional ethical codes (16, 17, 50) and fostering a socially responsible culture within organizations developing neurotechnologies (6, 9, 38). Support and incentives (6, 24, 35) should be provided to developers to encourage ethically responsible technologies and reward those who succeed (24, 35).

#### Interdisciplinary Collaboration

Interdisciplinary collaboration among researchers (5, 13, 15, 17, 18, 24, 29, 41, 45) and engagement with stakeholders (6, 24, 27, 38, 39, 40, 42, 45, 47, 51) were recognized as crucial for achieving ethical and responsible governance. Some sources raised the importance of culturally inclusive dialogues and incorporating diverse perspectives to tackle broader issues like equality and distributive justice (24, 27, 39, 47, 51). This openness to collaboration allows developers to leverage external ideas and engage with stakeholders (6, 29, 45), furthering the outcomes of ethical discourse (21, 38). Local interdisciplinary or inter-institutional collaborations could be leveraged to form international consortia (19, 25, 31, 35, 43, 48, 51) or lead to internationally accepted governance strategies (6, 16, 24, 47).

#### Public Engagement

Many articles emphasized the responsibility of developers to communicate findings to the public (15, 21) and engage them in dialogues) about neurotechnology and its societal impact (5, 6, 16, 17, 21, 22, 23, 40, 41, 51). Additionally, the public was regarded as an important voice in policy decisions (3, 16, 24, 31, 39) for their localized and diverse insight, and helping to determine what constitutes public benefit, with one article calling for empirical methods to assess public views on these ethical questions (24). This dialogue was seen as essential for building community trust, securing a social license for neurotechnological innovation, and demonstrating transparency (6, 13, 23, 51).

Educating the public on ethical issues and socio-technical solutions was encouraged (6, 16, 22, 23, 41, 45, 50). Training could also extend to specialists (e.g. rTMS technicians) (18) and health professionals who would benefit from ethics training (6, 13, 16, 24).

#### Scientific Integrity

Articles emphasized the importance of rigorous scientific practices (13,15, 23, 47). This included the importance of open science and open data sharing (3, 4, 6, 18, 21, 29, 42, 45), careful data collection (18) and management (15), reproducibility (21, 47) and the balanced reporting of results (5, 42). It was also suggested that clinicians and scientists avoid and disclose conflicts of interest (12, 37).

#### Epistemic Humility

Several sources elucidated the damage that unrealistic expectations and a lack of understanding about neurotechnologies can cause to the public and patients (2, 15, 40, 43, 48). The use of epistemic humility, such as providing a balanced portrayal of the likely benefits of using a technology (8, 11, 17, 21, 23, 29, 47, 49), clarifying the limits of current scientific knowledge (21, 49), anticipating and dispelling false narratives (5, 34), and identifying issues the public might find sensitive (23) were seen as critical to managing patient and public expectations. Misconceptions can lead to public disillusionment and rejection of neurotechnologies when they fail to meet unrealistic expectations (34, 40). Some articles emphasized the need to communicate alternative, non-technological solutions to health concerns (8, 11).

#### Neurorights

Concerns about brain manipulation and the collection and sharing of neural data threatening the privacy of thoughts and identities led to calls for “neurorights.” These neurorights, akin to the Universal Declaration of Human Rights, would establish new human rights specific to brain data and neurological manipulation (16, 20, 30, 35). Neurorights were primarily aimed to address issues of brain data privacy (20, 30, 35, 44, 47) and concerns about agency and identity (16, 20, 30).

Discussion on neurorights also raised philosophical concerns, such as free will (20) and freedom of thought (30, 47), which includes self-determination over one’s mind (30) and notably the right to be shielded from harmful manipulations of mental activity (30, 35, 47). One article argued against focusing narrowly on neurorights in favour of more comprehensive policies (40), while others concluded neurorights could enhance data protection (16, 30, 35, 47).

#### Legislation

Sources presented mixed views on the utility of legislation for the development and use of neurotechnologies. Some emphasized the necessity of legislation to ensure safe and responsible use (16, 40), while others warned that it could hinder innovation (34, 51). For instance, the European directive on minimum electromagnetic field exposure could restrict some MRI research (34). Some articles advocated for specific standards for various neurotechnological applications (5, 42) such as the *IEEE standards roadmap: neurotechnology for brain machine interfacing* (IEEE 2020). Broader legislative proposals included international conventions like the 2010 International Convention for the Protection of All Persons from Enforced Disappearance, aimed at defining and prohibiting certain actions related to neurotechnologies, though specific actions to be prohibited were not clearly defined (16).

## Discussion

Our analysis produced thirteen key themes emerging from the data, six of which were ethical themes and seven governance strategies. Nearly half of the articles covered prominent ethical themes, including justice, beneficence and nonmaleficence, social responsibility and accountability, and interdisciplinary collaboration. While these broader ethical themes were consistent throughout the documents, substantial variation was identified in the specific issues discussed and the response suggested to address these concerns. The identification of ethical themes and governance strategies in most publications was based on literature research and expert processes. People with lived experience were not involved in the derivation of ethical themes or governance strategies, as proposed by the Responsible Research and Innovation framework (von Schomberg 2013). As a result, it is possible that important ethical or regulatory issues have been overlooked.

### Underrepresentation and Ethical Homogeneity

Articles from low and middle income (LMIC’s) countries and the Global South were underrepresented. This suggests that existing guidelines are likely to reflect the priorities of High-Income Countries (HIC) and may fail to address those of LMICs. LMICs face unique social and historical challenges and have different priorities than those dominating current debates on responsible neurotechnology development in the Global North. For instance, while many articles discussed inequitable access, none addressed how high-cost interventions might divert funds from more basic healthcare needs, a significant consideration in LMICs where resources are limited (Stein and Giordano 2015). Furthermore, without local knowledge, the relevance and applicability of current ethical debates to researchers, developers, and practitioners in these nations may be compromised.

Additionally, almost half the articles were published by lead authors based in the United States. A focus on Anglo-U.S. bioethical approaches that prioritize individual rights and freedoms (Beauchamp and Childress 2019) may neglect alternative ethical frameworks such as Afro-ethical and Confucian moral values that emphasize collectivist cultures and values. As Awad and colleagues (2018) have shown, ethical principles can vary significantly across collectivist and individualist cultures, particularly regarding the value placed on individual autonomy and privacy (Segun 2021). This was a major weakness in current debates that may not capture a broader range of global perspectives such as those from the Global South.

Further the ethical issues we coded centred around themes of justice, autonomy, beneficence, and non-maleficence. These themes aligned closely with a principlist conception of ethics, despite principlism not being assumed prior to analysis. This suggests a strong principlist leaning in neuroethics scholarship, which may reflect that a large proportion of articles were published in the United States where principlism originated. Alternative approaches, such as virtue ethics and care ethics, are gaining traction in ethical scholarship (Pullen-Sansfacon 2010; Hamington 2019). Although these approaches were not entirely absent in the guidelines, for instance, the call to promote a culture of stewardship among developers (51), they were notably less prominent in the reviewed sources. The principlist leaning and the limited presence of alternative ethical frameworks may indicate a lack of philosophical diversity within neuroethics.

It is critical to acknowledge the limitations of the current neuroethical debates that are dominated by Anglo-U.S. perspectives and to amplify the views of those excluded from discussions on responsible innovation of neurotechnology. Future guidelines should aim to engage with alternative ethical approaches and promote a plurality of global viewpoints to ensure they represent a broader range of ethical perspectives.

### Scope and Actionability of Ethical Issues and Recommendations

Issues of justice emerged as the most frequently discussed ethical issue in the literature. This observation runs contrary to, but does not necessarily contradict, Matshabane’s (2021) critique, which argued that neuroethics agendas have been overly preoccupied with neurotechnological advancement, enhancement, and brain data. Given that Matshabane’s critique was published in 2021, it may have accurately reflected the state of the field at that time, as subsequent publications have increased their focus on justice related issues.

Matshabane (2021) was correct in suggesting that prominent neuroethics scholarship lacks detailed and practical governance on addressing justice and fails to discuss how justice should be upheld for diverse groups and communities. While accessibility and distributive justice were common concerns, including, for example, the poor historical record of the health system providing accessible services (9, 15, 16), articles failed to examine how this would be avoided or overcome in the innovation of neurotechnologies, many of which are extremely expensive and unfairly distributed. The lack of concrete recommendations or practical guidelines in this area was particularly notable compared to safety and privacy concerns, which were often met with regulatory suggestions. Future research needs to develop practical strategies to address justice concerns and how to address these ethical concerns in diverse groups and communities.

The themes autonomy, agency, identity, dignity, and moral status, referred to in eighteen articles, were relatively underdeveloped and vague; a view reflected in our consultation with experts. This may reflect the more speculative nature of these concerns about what neurotechnologies might be able to do. Advances in neurotechnologies challenge the traditional view of the “essence” of being human. They question the traditional humanistic view of “mind over matter,” positing that consciousness and self-awareness are directly tied to the biological functions of the brain. This challenges the long-held humanistic worldview, which places humans on a pedestal due to their distinct “dignity” (Benedikter et al. 2010). These challenges will need to be examined further in future governance documents.

The social responsibility of researchers and developers to ensure that their innovations provide benefit with minimal harm was widely recognized. There was, however, little detail on how responsibility should be enacted among industry professionals. For example, little detail was provided on how to incentivize or reprimand ethical conduct through binding governance strategies. Similarly, epistemic humility, that is to report your results in a balanced manner that acknowledges the limits of our current understanding, was often directed at researchers and developers. However, both researchers and commercial developers have incentives to inflate the implications of their research, including obtaining future funding and employment. Very little pragmatic guidance was provided on how to balance the need for responsible ethical innovation and pressing institutional and professional interests.

### Neuroexceptionalism and Speculative Ethics

Epistemic humility, particularly the caution not to exaggerate potential harms of neurotechnology was a widely supported governance strategy, proposed by fifteen articles. Many articles however, discussed ethical issues beyond the current capabilities and immediate concerns of neurotechnology. This was primarily driven by neuroexceptionalism: that is, the belief that brain data is inherently unique, privileged, or “true,” and by neurohype: which involves speculative ethical recommendations that exceed the technology’s present capabilities (Bublitz 2022). Speculation about the future of neurotechnology and its related concerns becomes problematic when it leads to overly restrictive governance proposals, which may be the case with the proposed “neurorights” (Fins 2022). These rights are viewed by their proponents as essential protections against technologies that could “read our minds” or manipulate our neural activity (Yuste 2023). However, these novel rights have drawn significant criticism for undermining established human rights and being poorly drafted (Bublitz 2022). Critics argue that assumptions about neurotechnologies’ ability to read minds are speculative at best, contributing to ethical “hype” (Gilbert and Russo 2024). It is crucial to distinguish between neurotechnologies reading minds and interpreting neural activity. Blurring this distinction can lead to exaggerated and speculative ethical decisions, disconnected from more grounded clinical and research concerns (Gilbert and Russo 2024). Future governance must stay rooted in technological realities and avoid hype that distorts the formation of clear, practical policies.

Documents offering guidelines concerning privacy and brain data were comprehensive, providing actionable strategies to protect privacy (38, 46, 47). However, this perhaps suggests an unjustified preoccupation with privacy in neurotechnology ethics, driven by neuroexceptionalism. Some commentators argued that people’s behaviour suggests that privacy is not as significant to them as some ethicists assume. Billions of social media users are willingly giving up their data and, by extension, their privacy for free access and personalized user experiences (Statista 2019). Therefore, it is not clear that people would value privacy as highly when it comes to neurotechnologies and brain profiles that offer even more powerful capabilities. Research is needed to explore community perceptions of brain privacy and its importance rather than rely on assumptions that are based on neuroexceptional accounts of brain research.

### Oversight and Underrepresentation

There was a noticeable lack of discussion around situations where ethical principles conflict. Although principlism was the dominant ethical framework in these guidelines, very little attention was given to one of its core challenges: how to resolve competing principles (Beauchamp and Childress 2019). For example, the tension between the right to privacy and the need for open data sharing to support scientific integrity was acknowledged but left unresolved. For organizations and policymakers to effectively implement or be guided by these principles, a clear method for prioritizing or balancing them is essential. Without this, stakeholders may resort to the most convenient solutions, potentially overlooking more critical ethical concerns.

There was a lack of comparisons with guidelines from related fields, such as AI ethics, which is more advanced in ethical scholarship with concrete initiatives being implemented (Berger and Rossi 2022). For example, the E.U. guidelines on AI ethics offer both soft and hard governance across all stages of AI design, development, and implementation (European Commission 2019). Few articles incorporated recommendations from such guidelines, most referring to theoretical scholarship to inform their frameworks. Drawing on established ethics guidelines from other fields like AI, could offer valuable insights for developing more effective frameworks.

Discussions and recommendations on the role of binding governance were limited, with most focusing on brain data. It would be beneficial for these articles to more clearly outline when binding governance, as opposed to soft law instruments should be applied. While binding governance such as laws and standardization has a valid role, this was rarely acknowledged. A clearer framework for determining when and where to apply soft law versus hard law instruments is a crucial area for future debate and development.

There was also a notable absence of discussion of animal ethics. Neuroscience research is highly dependent on animal research, including non-human primates with advanced cognitive capacities (Jones 2013). Only two sources (8, 22) referenced the ethical treatment of animals in research. This comes after recent news reports finding evidence that Neuralink’s primate subjects were euthanized after suffering complications like “bloody diarrhoea, partial paralysis, and cerebral edema” (Roth 2023, ¶1). Further guidelines should consider anthropocentric approaches and rethink how animals can be incorporated in the current guidelines. This shift will help address the growing ethical challenges posed by technological and scientific advancements involving nonhuman and engineered organisms (Johnson 2020).

### Limitations and Future Directions

This study had several limitations. The guidelines and frameworks analysed were exclusively from academic journals. Many published guidelines, frameworks, and policy documents from non-academic sources, such as governments and global regulatory institutions (OECD 2019; UNESCO 2024; IEEE Standards Association 2020), were not included. Future research should examine ethical guidance from these organizations. Additionally, this study was limited to articles published in English. Ethical guidelines and frameworks written in other languages may not have been identified, potentially skewing the geographic distribution of data. Future research should examine guidelines published in other major languages, such analysis is resource-intensive and beyond the scope of this study.

## Conclusion

This study provided an in-depth exploration of the ethical issues and strategies in neuroscience ethics literature related to neurotechnologies. We identified points of convergence and consensus among guidelines regarding ethical issues and strategies. The study highlighted the need for future research to address the lack of cultural and stakeholder diversity, underrepresented issues, and the unsubstantiated discourse on neurorights. Additionally, we suggested areas where these guidelines could be further developed to be more practical. These findings can help developers, research organizations, ethicists, and policymakers create a more comprehensive and universal set of governance documents.

## Supplementary Information

Below is the link to the electronic supplementary material.

Supplementary file1 (DOCX 56.7 KB)

## Data Availability

The data supporting this scoping review were obtained from publicly available published sources. All ethical guidelines and frameworks analysed are cited in the reference list and indexed in Supplementary Material 1.
