# Supplementary material for: Ethical Governance Strategies for the Responsible Innovation of Neurotechnologies: A Scoping Review
Source: J Bioeth Inq. 2025 Nov 11;22(4):931–48. doi: 10.1007/s11673-025-10440-9 (PMC12783205; doi:10.1007/s11673-025-10440-9)
Supplement: Supplementary file 1 — Supplementary file1 (DOCX 56.7 KB) [file 11673_2025_10440_MOESM1_ESM.docx]

**Supplementary Material 1**

| **Assigned Number** | **Title** | **Reference** |
| --- | --- | --- |
| 1 | A Just Standard The Ethical: A Just Standard: The Ethical Management of Incidental Findings in Brain Imaging Research | Graham, M., N. Hallowell and J. Savulescu (2021). "A Just Standard: The Ethical Management of Incidental Findings in Brain Imaging Research." Journal of Law, Medicine and Ethics 49(2): 269-281. |
| 2 | A marathon, not a sprint – neuroimaging, Open Science and ethic: A marathon, not a sprint – neuroimaging, Open Science and ethics | Beauvais, M. J. S., Knoppers, B. M., & Illes, J. (2021). A marathon, not a sprint - neuroimaging, Open Science and ethics. NeuroImage, 236, 118041. https://doi.org/10.1016/j.neuroimage.2021.118041 |
| 3 | A Neuroethics Backbone for the Evolving: A Neuroethics Backbone for the Evolving Canadian Brain Research Strategy | Illes, J., S. Weiss, J. Bains, J. A. Chandler, P. Conrod, Y. De Koninck, L. K. Fellows, D. Groetzinger, E. Racine, J. M. Robillard and M. B. Sokolowski (2019). "A Neuroethics Backbone for the Evolving Canadian Brain Research Strategy." Neuron 101(3): 370-374. |
| 4 | A principled and cosmopolitan neuroethics: A principled and cosmopolitan neuroethics: considerations for international relevance | Shook, J. R. and J. Giordano (2014). "A principled and cosmopolitan neuroethics: considerations for international relevance." Philosophy, Ethics and Humanities in Medicine : PEHM 9: 1. |
| 5 | A Surety Engineering: A surety engineering framework and process to address ethical, legal, and social issues for neurotechnologies | Shaneyfelt, W. L. and D. E. Peercy (2012). A surety engineering framework and process to address ethical, legal, and social issues for neurotechnologies. Neurotechnology: Premises, Potential, and Problems: 213-232. |
| 6 | Addressing neuroethics issues in practice: Addressing neuroethics issues in practice: Lessons learnt by tech companies in AI ethics | Berger, S. E., & Rossi, F. (2022). Addressing neuroethics issues in practice: Lessons learnt by tech companies in AI ethics. Neuron, 110(13) 2052-2056. https://doi.org/10.1016/j.neuron.2022.05.006 |
| 7 | An Equity and Justice-Informed Ethical Framework to Guide Incidental Findings in Brain Imaging Research: An Equity and Justice-Informed Ethical Framework to Guide Incidental Findings in Brain Imaging Research | Bhaskar, S. M. M. (2023). An Equity and Justice-Informed Ethical Framework to Guide Incidental Findings in Brain Imaging Research [Article]. Clinics and Practice, 13(1), 116-124. https://doi.org/10.3390/clinpract13010011 |
| 8 | Asilomar survey researcher perspectives on ethical principles and guidelines for BCI research: Asilomar survey: researcher perspectives on ethical principles and guidelines for BCI research | Pham, M., Goering, S., Sample, M., Huggins, J. E., & Klein, E. (2018). Asilomar survey: researcher perspectives on ethical principles and guidelines for BCI research. Brain-Computer Interfaces, 5(4), 97-111. https://doi.org/10.1080/2326263x.2018.1530010 |
| 9 | Building a culture of responsible neurotech: Building a culture of responsible neurotech: Neuroethics as socio-technical challenges | Robinson, J. T., Rommelfanger, K. S., Anikeeva, P. O., Etienne, A., French, J., Gelinas, J., Grover, P., & Picard, R. (2022). Building a culture of responsible neurotech: Neuroethics as socio-technical challenges. Neuron, 110(13) 2057-2062. https://doi.org/10.1016/j.neuron.2022.05.005 |
| 10 | Clinical Neuroinnovation Ethical: Clinical Neuroinnovation: Ethical Frameworks and Emerging Issues | Kasun, M., Dunn, L. B., Palmer, B. W., Kim, J. P., & Roberts, L. W. (2023). Clinical Neuroinnovation: Ethical Frameworks and Emerging Issues. In Ethics and Clinical Neuroinnovation: Fundamentals, Stakeholders, Case Studies, and Emerging Issues (pp. 57-79). https://doi.org/10.1007/978-3-031-14339-7_3 |
| 11 | Cognitive Enhancement and Beyond Recommendations from the Bioethics Commission: Cognitive Enhancement and Beyond: Recommendations from the Bioethics Commission | Allen, A. L., & Strand, N. K. (2015). Cognitive Enhancement and Beyond: Recommendations from the Bioethics Commission. Trends in cognitive sciences, 19(10), 549–551. https://doi.org/10.1016/j.tics.2015.08.001 |
| 12 | Consensus on guidelines for stereotactic: Consensus on guidelines for stereotactic neurosurgery for psychiatric disorders | Nuttin, B., Wu, H., Mayberg, H., Hariz, M., Gabriëls, L., Galert, T., Merkel, R., Kubu, C., Vilela-Filho, O., Matthews, K., Taira, T., Lozano, A. M., Schechtmann, G., Doshi, P., Broggi, G., Reǵis, J., Alkhani, A., Sun, B., Eljamel, S., . . . Schlaepfer, T. (2014). Consensus on guidelines for stereotactic neurosurgery for psychiatric disorders [Article]. Journal of Neurology, Neurosurgery and Psychiatry, 85(9), 1003-1008. https://doi.org/10.1136/jnnp-2013-306580 |
| 13 | Deep Brain Stimulation as Clinical Innovation: Deep brain stimulation as clinical innovation: An ethical and organizational framework to sustain deliberations about psychiatric deep brain stimulation | Bell, E., Leger, P., Sankar, T., & Racine, E. (2016). Deep brain stimulation as clinical innovation: An ethical and organizational framework to sustain deliberations about psychiatric deep brain stimulation [Review]. Neurosurgery, 79(1), 3-9. https://doi.org/10.1227/NEU.0000000000001207 |
| 14 | DUAL USE IN NEUROSCIENTIFIC: DUAL USE IN NEUROSCIENTIFIC AND NEUROTECHNOLOGICAL RESEARCH: A NEED FOR ETHICAL ADDRESS AND GUIDANCE | Giordano, J., & Evers, K. (2019). DUAL USE IN NEUROSCIENTIFIC AND NEUROTECHNOLOGICAL RESEARCH: A NEED FOR ETHICAL ADDRESS AND GUIDANCE. In Z. Koporc (Ed.), Ethics and Integrity in Health and Life Sciences Research (Vol. 4, pp. 129-145). https://doi.org/10.1108/s2398-601820180000004009 |
| 15 | Ethics-guidance-for-neurological-and-ps_2013_Handbook-of-Clinic: Ethics guidance for neurological and psychiatric deep brain stimulation | Bell, E., & Racine, E. (2013). Ethics guidance for neurological and psychiatric deep brain stimulation [Research Support, Non-U.S. Gov't |
| 16 | Four ethical priorities for neurotechnologies and AI: Four ethical priorities for neurotechnologies and AI | Yuste, R., Goering, S., Arcas, B. A. Y., Bi, G., Carmena, J. M., Carter, A., Fins, J. J., Friesen, P., Gallant, J., Huggins, J. E., Illes, J., Kellmeyer, P., Klein, E., Marblestone, A., Mitchell, C., Parens, E., Pham, M., Rubel, A., Sadato, N., . . . Wolpaw, J. (2017). Four ethical priorities for neurotechnologies and AI. Nature, 551(7679), 159-163. https://doi.org/https://dx.doi.org/10.1038/551159a |
| 17 | From Responsible Research and Innovation to responsibility by design: From Responsible Research and Innovation to responsibility by design | Stahl, B. C., Akintoye, S., Bitsch, L., Bringedal, B., Eke, D., Farisco, M., Grasenick, K., Guerrero, M., Knight, W., Leach, T., Nyholm, S., Ogoh, G., Rosemann, A., Salles, A., Trattnig, J., & Ulnicane, I. (2021). From Responsible Research and Innovation to responsibility by design [Article]. Journal of Responsible Innovation, 8(2), 175-198. https://doi.org/10.1080/23299460.2021.1955613 |
| 18 | From vision to action: From vision to action: Canadian leadership in ethics and neurotechnology | Illes, J., Lipsman, N., McDonald, P. J., Hrincu, V., Chandler, J., Fasano, A., Giacobbe, P., Hamani, C., Ibrahim, G. M., Kiss, Z., Meng, Y., Sankar, T., & Weise, L. (2021). From vision to action: Canadian leadership in ethics and neurotechnology [Research Support, N.I.H., Extramural |
| 19 | Insight into Neuroethical Considerations of the Newly Emerging: Insight into Neuroethical Considerations of the Newly Emerging Technologies and Techniques of the Global Brain Initiatives | Wood, C. R., Xi, Y., Yang, W. J., & Wang, H. (2023). Insight into Neuroethical Considerations of the Newly Emerging Technologies and Techniques of the Global Brain Initiatives [Note]. Neuroscience Bulletin, 39(4), 685-689. https://doi.org/10.1007/s12264-022-00984-w |
| 20 | International data governance for neuroscience: International data governance for neuroscience | Eke, D. O., Bernard, A., Bjaalie, J. G., Chavarriaga, R., Hanakawa, T., Hannan, A. J., Hill, S. L., Martone, M. E., McMahon, A., Ruebel, O., Crook, S., Thiels, E., & Pestilli, F. (2022). International data governance for neuroscience [Review]. Neuron, 110(4), 600-612. https://doi.org/10.1016/j.neuron.2021.11.017 |
| 21 | neuroethical-responsibilities: Neuroethical responsibilities | Racine, E. and J. Illes (2006). "Neuroethical responsibilities." Canadian Journal of Neurological Sciences 33(3): 269-277. |
| 22 | Neuroethics for the National Institutes of Health BRAIN initiative: Neuroethics for the national institutes of health BRAIN initiative | Bianchi, D. W., Cooper, J. A., Gordon, J. A., Heemskerk, J., Hodes, R., Koob, G. F., Koroshetz, W. J., Shurtleff, D., Sieving, P. A., Volkow, N. D., Churchill, J. D., & Ramos, K. M. (2018). Neuroethics for the National Institutes of Health BRAIN Initiative. The Journal of neuroscience : the official journal of the Society for Neuroscience, 38(50), 10583–10585. https://doi.org/10.1523/JNEUROSCI.2091-18.2018 |
| 23 | Neuroethics guiding principles for the NIH BRAIN Initiative: Neuroethics guiding principles for the NIH BRAIN Initiative | Greely, H. T., Grady, C., Ramos, K. M., Chiong, W., Eberwine, J., Farahany, N. A., Johnson, L. S. M., Hyman, B. T., Hyman, S. E., Rommelfanger, K. S., & Serrano, E. E. (2018). Neuroethics Guiding Principles for the NIH BRAIN Initiative. The Journal of neuroscience : the official journal of the Society for Neuroscience, 38(50), 10586–10588. https://doi.org/10.1523/JNEUROSCI.2077-18.2018 |
| 24 | Neuroethics Questions to Guide Ethical: Neuroethics Questions to Guide Ethical Research in the International Brain Initiatives | Amadio, J., B. Guo-Qiang, P. F. Boshears, A. Carter, A. Devor, K. Doya, H. Garden, J. Illes, L. S. M. Johnson, L. Jorgenson, J. Bang-Ook, I. Lee, P. Michie, T. Miyakawa, E. Nakazawa, O. Sakura, H. Sarkissian, S. Laura Specker, S. Uh, D. Winickoff, W. Paul Root, W. Kevin Chien-Chang, A. Yasamura, J. C. Zheng, K. S. Rommelfanger, J. Sung-Jin, E. Arisa, T. Fukushi, K. Kasai, K. M. Ramos, A. Salles and I. Singh (2018). "Neuroethics Questions to Guide Ethical Research in the International Brain Initiatives." Neuron 100(1): 19-36. |
| 25 | Neuroimaging and Disorders of Consciousness: Neuroimaging and disorders of consciousness: Envisioning an ethical research agenda | Fins, J. J., J. Illes, J. L. Bernat, J. Hirsch, S. Laureys and E. Murphy (2008). Neuroimaging and disorders of consciousness: Envisioning an ethical research agenda. American Journal of Bioethics. |
| 26 | Neurostimulation Devices for Cognitive Enhancement Toward a Comprehensive Regulatory Framework: Neurostimulation Devices for Cognitive Enhancement: Toward a Comprehensive Regulatory Framework | Dubljevic, V. (2015). "Neurostimulation Devices for Cognitive Enhancement: Toward a Comprehensive Regulatory Framework." Neuroethics 8(2): 115-126. |
| 27 | Neurotech-Ethics Suggestions for the Way Forward: Neurotech-ethics: Suggestions for the way forward | Coenen, C. and T. Stieglitz (2021). Neurotech-ethics: Suggestions for the way forward. International IEEE/EMBS Conference on Neural Engineering, NER. |
| 28 | Neurotechnological assessment of: Neurotechnological assessment of consciousness disorders: Five ethical imperatives | Evers, K. (2016). "Neurotechnological assessment of consciousness disorders: Five ethical imperatives." Dialogues in Clinical Neuroscience 18(2): 155-162. |
| 29 | Neurotechnology and Society Strengthening: Neurotechnology and Society: Strengthening Responsible Innovation in Brain Science | Garden, H., D. M. Bowman, S. Haesler and D. E. Winickoff (2016). "Neurotechnology and Society: Strengthening Responsible Innovation in Brain Science." Neuron 92(3): 642-646. |
| 30 | On Neurorights: On Neurorights | Ienca, M. (2021). "On Neurorights." Frontiers in Human Neuroscience. |
| 31 | Policy Analysis for Implementing Neuroethics in Korea’s Brain Research Promotion Act: Policy Analysis for Implementing Neuroethics in Korea’s Brain Research Promotion Act | Kang, T. W., T. W. Oh and S. J. Jeong (2023). "Policy Analysis for Implementing Neuroethics in Korea?s Brain Research Promotion Act." Experimental Neurobiology 32(1): 1-7. |
| 32 | Practical approaches to incidental findings in brain imaging research: Practical approaches to incidental findings in brain imaging research | Illes, J., M. P. Kirschen, E. Edwards, P. Bandettini, M. K. Cho, P. J. Ford, G. H. Glover, J. Kulynych, R. MacKlin, D. B. Michael, S. M. Wolf, T. Grabowski and B. Seto (2008). "Practical approaches to incidental findings in brain imaging research." Neurology 70(5): 384-390. |
| 33 | Proceedings of the Fourth Annual Deep Brain Stimulation Think Tank A Review of Emerging Issues and Technologies: Proceedings of the Fourth Annual Deep Brain Stimulation Think Tank: A Review of Emerging Issues and Technologies | Deeb, W., Giordano, J. J., Rossi, P. J., Mogilner, A. Y., Gunduz, A., Judy, J. W., Klassen, B. T., Butson, C. R., Van Horne, C., Deny, D., Dougherty, D. D., Rowell, D., Gerhardt, G. A., Smith, G. S., Ponce, F. A., Walker, H. C., Bronte-Stewart, H. M., Mayberg, H. S., Chizeck, H. J., Langevin, J. P., … Okun, M. S. (2016). Proceedings of the Fourth Annual Deep Brain Stimulation Think Tank: A Review of Emerging Issues and Technologies. Frontiers in integrative neuroscience, 10, 38. https://doi.org/10.3389/fnint.2016.00038 |
| 34 | Prospecting responsible technology paths: Management options for an appropriate societal embedding of medical neuroimaging: Prospecting responsible technology paths: Management options for an appropriate societal embedding of medical neuroimaging' | Arentshorst, M. E., T. D. Buning, W. P. C. Boon and J. E. W. Broerse (2015). "'Prospecting responsible technology paths: Management options for an appropriate societal embedding of medical neuroimaging'." Science and Public Policy 42(6): 775-788. |
| 35 | Recommendations for responsible development and application of neurotechnologies: Recommendations for responsible development and application of neurotechnologies | Goering, S., E. Klein, L. Specker Sullivan, A. Wexler, B. Aguera y Arcas, G. Bi, J. M. Carmena, J. J. Fins, P. Friesen, J. Gallant, J. E. Huggins, P. Kellmeyer, A. Marblestone, C. Mitchell, E. Parens, M. Pham, A. Rubel, N. Sadato, M. Teicher, D. Wasserman, M. Whittaker, J. Wolpaw and R. Yuste (2021). "Recommendations for responsible development and application of neurotechnologies." Neuroethics 14(3): 365-386. |
| 36 | Regulating the Use of Cognitive Enhancement an Analytic Framework: Regulating the Use of Cognitive Enhancement: an Analytic Framework | Jwa, A. S. (2019). "Regulating the Use of Cognitive Enhancement: an Analytic Framework." Neuroethics 12(3): 293-309. |
| 37 | Responding to requests from adult patients: Responding to request from adult patients for neuroenhancements. Guidance of the Ethics, Law, and Humanities Committee | Larriviere, D., Williams, M. A., Rizzo, M., Bonnie, R. J., & AAN Ethics, Law and Humanities Committee (2009). Responding to requests from adult patients for neuroenhancements: guidance of the Ethics, Law and Humanities Committee. Neurology, 73(17), 1406–1412. https://doi.org/10.1212/WNL.0b013e3181beecfe |
| 38 | Responsible big data governance: Responsible data governance of neuroscience big data | Fothergill, B. T., Knight, W., Stahl, B. C., & Ulnicane, I. (2019). Responsible Data Governance of Neuroscience Big Data. Frontiers in neuroinformatics, 13, 28. https://doi.org/10.3389/fninf.2019.00028 |
| 39 | Responsible Research and Innovation in the context of human cognitive enhancement some essential features: Responsible Research and Innovation in the context of human cognitive enhancement: some essential features | Shelley-Egan, C., A. B. Hanssen, L. Landeweerd and B. Hofmann (2018). "Responsible Research and Innovation in the context of human cognitive enhancement: some essential features." Journal of Responsible Innovation 5(1): 65-85. |
| 40 | Rethinking the ethical priorities for brain–computer interfaces: Rethinking the ethical priorities for brain–computer interfaces | Cabrera, L. Y. and D. J. Weber (2023). "Rethinking the ethical priorities for brain–computer interfaces." Nature Electronics 6(2): 99-101. |
| 41 | Social impact and governance of AI and neurotechnologies: Social impact and governance of AI and neurotechnologies | Doya, K., A. Ema, H. Kitano, M. Sakagami and S. Russell (2022). "Social impact and governance of AI and neurotechnologies." Neural Networks 152: 542-554. |
| 42 | Standardization of Neurotechnology for: Standardization of neurotechnology for brain-machine interfacing: State of the art and recommendations | Chavarriaga, R., C. Carey, J. L. Contreras-Vidal, Z. McKinney and L. Bianchi (2021). "Standardization of neurotechnology for brain-machine interfacing: State of the art and recommendations." IEEE Open Journal of Engineering in Medicine and Biology 2: 71-73. |
| 43 | The Asilomar Survey Stakeholders’ Opinions on Ethical Issues related to BrainComputer Interfacing: The Asilomar Survey: Stakeholders’ Opinions on Ethical Issues related to Brain-Computer Interfacing | Nijboer, F., Clausen, J., Allison, B. Z., & Haselager, P. (2013). The Asilomar Survey: Stakeholders' Opinions on Ethical Issues Related to Brain-Computer Interfacing. Neuroethics, 6(3), 541–578. https://doi.org/10.1007/s12152-011-9132-6 |
| 44 | The ethical and legal landscape of brain data governance: The ethical and legal landscape of brain data governance | Ochang, P., Stahl, B. C., & Eke, D. (2022). The ethical and legal landscape of brain data governance. PloS one, 17(12), e0273473. https://doi.org/10.1371/journal.pone.0273473 |
| 45 | The Human Brain Project Responsible: The Human Brain Project: Responsible Brain Research for the Benefit of Society | Salles, A., Bjaalie, J. G., Evers, K., Farisco, M., Fothergill, B. T., Guerrero, M., Maslen, H., Muller, J., Prescott, T., Stahl, B. C., Walter, H., Zilles, K., & Amunts, K. (2019). The Human Brain Project: Responsible Brain Research for the Benefit of Society. Neuron, 101(3), 380–384. https://doi.org/10.1016/j.neuron.2019.01.005 |
| 46 | The spectrum of data sharing policies in neuroimaging data repositories: The spectrum of data sharing policies in neuroimaging data repositories | Jwa, A. S., & Poldrack, R. A. (2022). The spectrum of data sharing policies in neuroimaging data repositories. Human brain mapping, 43(8), 2707–2721. https://doi.org/10.1002/hbm.25803 |
| 47 | Towards a Governance Framework for Brain Data: Towards a governance framework for brain data | Ienca, M., J. J. Fins, R. J. Jox, F. Jotterand, S. Voeneky, R. Andorno, T. Ball, C. Castelluccia, R. Chavarriaga, H. Chneiweiss, A. Ferretti, O. Friedrich, S. Hurst, G. Merkel, F. Molnar-Gabor, J.-M. Rickli, J. Scheibner, E. Vayena, R. Yuste and P. Kellmeyer (2022). "Towards a governance framework for brain data." Neuroethics 15: 1-14. |
| 48 | Towards responsible neuroimaging applications in health care: Towards responsible neuroimaging applications in health care: Guiding visions of scientists and technology developers | Arentshorst, M. E., J. E. W. Broerse, A. Roelofsen and T. De Cock Buning (2014). Towards responsible neuroimaging applications in health care: Guiding visions of scientists and technology developers. Responsible Innovation 1: Innovative Solutions for Global Issues: 255-280. |
| 49 | Transcranial magnetic stimulation deep brain stimulation and personal identity Ethical questions, and neuroethical approaches for medical practice: | Jotterand, F. and J. Giordano (2011). "Transcranial magnetic stimulation, deep brain stimulation and personal identity: Ethical questions, and neuroethical approaches for medical practice." International Review of Psychiatry 23(5): 476-485. |
| 50 | Why Neuroscientists: Why neuroscientists should take the pledge: A collective approach to the misuse of neuroscience | Bell, C. (2014). Why neuroscientists should take the pledge: A collective approach to the misuse of neuroscience. Neurotechnology in National Security and Defense: Practical Considerations, Neuroethical Concerns: 227-238. |
| 51 | The OECD Approach to Responsible Innovation: The OECD Approach to Responsible Innovation | Winickoff, D. E. and H. Garden (2021). The OECD Approach to Responsible Innovation. Convergence Mental Health: A Transdisciplinary Approach to Innovation. E. Smith, H. Lavretsky, C. F. Reynolds Iii et al., Oxford University Press: 0. |
